# Multivisceral and Small Bowel Transplantation at Shiraz Organ Transplant Center

**Published:** 2012-05-01

**Authors:** S. Nikeghbalian, S. H. Mehdi, M. Aliakbarian, K. Kazemi, A. Shamsaeefar, A. Bahreini, M. R. Mansoorian, S. A. Malekhosseini

**Affiliations:** 1 *Shiraz Transplant Research Center, Shiraz University of Medical Sciences, Shiraz, Iran,*; 2 *Surgical Oncology Research Center, Imam Reza Hospital, Faculty of Medicine, Mashhad University of Medical Sciences, Mashhad, Iran*

**Keywords:** Multivisceral, Modified multivisceral, Small bowel, Transplantation, Animal model, Abdominal malignancies

## Abstract

Background: Multivisceral transplantations were initially done in animal models to understand the immunological effects. Later on, in human beings, it has been considered a salvage procedure for unresectable complex abdominal malignancies. With advancement in surgical techniques, availability of better immunosuppressive drugs, and development of better post-operative management protocols, outcomes have been improved after these complex surgical procedures.

Objective: To analyze and report results of multivisceral, modified multivisceral, and small bowel transplantations done at Shiraz Organ Transplant Center, Shiraz, southern Iran.

Methods: Medical records of all patients who underwent multivisceral, modified multivisceral, and small bowel transplants were retrospectively analyzed.

Results: There were 18 patients. The most common indications for the procedure in our series were unresectable carcinoma of pancreas followed by short bowel syndrome. 10 patients were alive after a median follow-up of 8.7 (range: 3–32) months. The remaining 8 patients died post-operatively, mostly from septicemia.

Conclusion: Multivisceral and small bowel transplantations are promising treatments for complex abdominal pathologies.

## INTRODUCTION

The first multivisceral transplantation (MVTx) was done by Thomas Starzl, *et al*, more than 50 years ago in animal model to study the immunological effects with no intention for clinical application [1]. Lillehei, *et al*, also study the procedure in canine model in the same era [2]. In mid-1980s, Starzl group performed the first human multivisceral transplantation, however, it was associated with marked surgical and immunological complications. Their first patients died shortly after transplantations [3]. 

Modern era of MVTx developed based on those pioneering works and became feasible when tacrolimus was introduced as an immunosuppressive agent in 1989. Various modifications were then introduced; in classical multivisceral transplantation, liver along with stomach, intestine and pancreaticoduodenal complex (with or without spleen) are transplanted concurrently, while in modified multivisceral transplantation (MMVTx) the graft does not include liver [4]. Patients who are candidates for MVTx or MMVTx usually have no other surgical or medical treatment options [5]. Modified multivisceral and intestinal transplants are indicated in patients with intestinal failure before developing total parenteral nutrition (TPN)-associated liver failure. A liver biopsy is mandatory even if clinical and biochemical parameters show no signs of liver disease as only TPN-related cholestasis is reversible after intestinal transplantation [6, 7].

Recent advances in the surgical techniques as well as availability of more potent immunosuppressive drugs have led to improved survival. Although the incidence of PTLD have been increased as a side-effect of more immunosuppression, long-term outcome in some studies revealed that preservation of native spleen may prevent development of PTLD [8, 9].

We conducted this study to determine the outcome of all patients who received small bowel or MVTx at Shiraz Organ Transplant Center, Shiraz, southern Iran, between June 2010 and December 2012.

## MATERIAL AND METHODS

During the mentioned period, 18 patients underwent MVTx, MMVTx, and small bowel transplantation. Among them, eight had classical MVTx, four MMVTx, four isolated small bowel transplants, and two had combined pancreas and small bowel transplants.

Indications for performing the procedure can be categorized into three groups (Table 1):

**Table 1:** Characteristics of recipients

Present condition	Follow-up duration (month)	Treat Group*	Tx	Pre-operative diagnosis	Sex	Age
Alive	32	2	MVTx^†^	Desmoid tumor	M	30
Exp	4	3	MVTx	Hepatocellular carcinoma	M	35
Alive	4	2	MVTx	Neuroendocrine tumor	M	14
Exp	6	2	MMVTx^‡^	Gastrointestinal stromal tumor	F	35
Exp	5	3	MVTx	Small bowel insufficiency following ex-vivo resection of pancreatic carcinoma	M	47
Alive	3	3	MVTx	Carcinoma of the pancreas	M	30
Alive	2	1	MMVTx	Chronic rejection of first pancreas transplant	F	40
Alive	17	1	MMVTx	Abdominal trauma	M	50
Exp	4	3	MMVTx	Carcinoma of the pancreas	M	39
Exp	18	1	Small intestine	Bowel gangrene	F	50
Alive	13	1	Small intestine	Abdominal trauma	M	46
Exp	10	2	MMVTx	Gastrointestinal stromal tumor	F	55
Exp	10	2	MVTx	Neuroendocrine tumor	F	32
Alive	9	1	Small intestine	Bowel gangrene	M	43
Alive	7	1	Small intestine	Bowel gangrene	M	34
Alive	6	1	MVTx	Liver cirrhosis with extensive PV and SMV thrombosis	M	51
Exp	3	3	MVTx	Small round cell tumor	M	26
Alive	3	1	MMVTx	Short bowel syndrome due to venous thrombosis	F	29

* Treatment group: For this classification see the text

† Multivisceral transplantation

‡ Modified multivisceral transplantation

Patients with benign processes which included short bowel syndrome, liver cirrhosis with extensive venous thrombosis and abdominal trauma (n=8). Moreover, in this group there was a case of pancreas transplant who developed chronic rejection of the transplanted pancreas and thrombosis in superior mesenteric vein for whom MMVTx (including small bowel and pancreaticoduodenal complex) was done.

Transplantation for low malignant potential tumors, which are usually slow-growing, including desmoid, neuroendocrine, and gastrointestinal tumors (n=5).

The last group included locally advanced malignancies that were not resectable with conventional techniques, including pancreatic carcinoma and hepatocellular carcinoma (n=5). There were some common characteristics in this group:

All cases were young people. 

They had locally advanced tumors which were unresectable because of anatomical limitations like SMA involvement without any evidence of distant metastases.

R0 resection was possible with *en-bloc* resection and MVTx.

One of the patients in this group underwent *ex vivo* resection of the pancreatic adenocarcinoma and small bowel autotransplantation and needed MVTx because of small bowel insufficiency three months after the first procedure. Another case underwent MVTx due to hilar involvement in a patient with hepatocellular carcinoma.

All donors were deceased and had a mean±SD age of 26±10.05 years. The most common cause of brain death was trauma (78%); all patients received ABO-identical grafts. Lymphocyte cross-match was done in all cases and transplants were done only in the presence of negative lymphocyte cross-match. Immunomodulation was not done pre-operatively for patients. 

Harvesting was done as *en-bloc* procedure in all cases and any organ not needed in the procedure removed at back table dissection. Except in one case in whom split right lobe was used, whole liver grafts were used in classical MVTx.

All patients were induced with alemtuzumab (Campath 1H). Maintenance immunosuppression included tacrolimus (trough level 12–15 ng/mL), mycophenolate mofetil 30 mg/kg/day and low dose steroids. Sirolimus was added in patients who developed renal dysfunction to reduce the dose of tacrolimus or to boost immunosuppression in patients with rejection episodes. Rejection episodes were treated with increasing dose of immunosuppressives, using high dose steroids or using biological agents, depending on the grade and severity of the episode.

All patients received prophylaxis against bacterial, fungal and viral infections. Episodes of infection were treated with appropriately according to culture and sensitivity reports.

Routine intestinal biopsies were taken through ileostomy stoma twice weekly for the first three weeks, followed by weekly for the next two months, and monthly afterward. In case of suspected rejection episode, biopsies were taken more frequently. 

Intravenous feeding was started immediately in all patients after transplantation; it was followed by enteral feeding via jejunostomy tube. Enteral feeding was started with simple elemental formulae and slowly increased in both quantity and strength to full diet as tolerated by the patient.

Recipient surgery 

In classical MVTx, suprahepatic IVC was first anastomosed to the recipients’ hepatic veins. The donor’s abdominal aorta containing both celiac artery and superior mesenteric arteries were then anastomosed to the recipient infrarenal aorta in end-to-side fashion. In case of MMVTx, portal vein of graft was anastomosed to the portal vein of the recipient in end-to-end fashion.

Reconstruction of gastrointestinal system also depends on the type of transplantation. In case of classical MVTx and MMVTx, proximal anastomosis is performed between native esophagus and anterior wall of the stomach with pyloroplasty while distal end of the graft is exteriorized as end stoma after creating side-to-side ileocolic anastomosis. In isolated intestinal transplantation, proximal anastomosis was made by duodenojejunostomy between recipient duodenum and graft jejunum. At the end, a jejunostomy tube was placed for enteral feeding.

## RESULTS

Between June 2010 and December 2012, we performed 18 MVTx and small bowel transplantation in our center. Out of these, eight underwent classical MVTx, four MMVTx, four isolated small bowel transplantation, and two had combined pancreas and small bowel transplantation. All patients were adults except one who was a 14-year-old male. The mean±SD age of patients was 38.1±10.6 years. Twelve patients were male and six were female. Indications for transplantation are shown in Table 1. 

Total procedure took between 450 and 600 minutes. Total cold and warm ischemia time ranged from 130–720, and 30–90 minutes, respectively. The mean hospital stay was 41.4 (range: 22–64) days. 

Complications following transplantations included major infection in five patients—one patient had large intra-abdominal collection, another had brain abscess, and three had severe sepsis. Other major complications were rejection episodes in seven patients, and GVHD in another patient. Recurrence of disease was not observed in our patients during the follow-up. Out of seven patients who experienced rejection episodes, one had mild grade 1 rejection and was treated with high dose steroids. In remaining six patients, the rejection was moderate to severe (grade 2 and 3) and was treated with ATG monoclonal antibody. One patient did not respond to any rejection treatments and died of severe rejection. GVHD was observed in only one patient in our series and managed by ATG monoclonal antibody.

Among the 18 patients, 10 were alive with a median follow-up of 8.7 (range: 3–32) months. Eight patients died during the follow-up. Causes of death were severe sepsis in five patients; the remaining three died secondary to primary non-function of the liver, DVT and pulmonary emboli, and severe rejection. 

## DISCUSSION

In the past 20 years, MVTx has evolved slowly and it is now considered a therapeutic option for patients with irreversible intestinal failure as well as unresectable complex abdominal pathologies [10-12]. The success rate of this procedure varies from center to center and depends on experience of the surgical team [13, 14]. 

There are several indications for MVTx and small bowel transplantation. After liver cirrhosis, portal vein thrombosis is the most common cause of portal hypertension. The major causes of non-cirrhotic portal hypertension include hypercoagulable states leading to spontaneous thrombosis, radiation, severe pancreatitis and intra-abdominal tumors [15]. In the presence of cirrhosis with portal vein thrombosis, liver transplantation can still be done in combination with thrombectomy or use of intervening jump graft. Nonetheless, in the presence of complete thrombosis of splanchnic circulation, the only viable option left is MVTx. Out of 18 patients we transplanted, one underwent MVTx for extensive splanchnic venous thrombosis. 

Starzl, *et al*, did the first MVTx for unresectable abdominal malignancies. However, their results were very poor. On account of the recent advancements in technique and immunosuppression and use of strict selection criteria, the survival rate of patients undergoing MVTx becomes as high as 77% [16, 17]. Intra-abdominal malignancies constituted the most common indication for MVTx or small bowel transplantation in our series. 

Trauma to abdomen is one of the known causes of severe combined solid organs and hollow visceral injuries that may result in chronic debilitating syndromes such as intestinal failure and pancreatic failure. TPN therapy is an option for survival in these patients but chronic TPN therapy can lead to life-threatening complications such as liver failure and line-related sepsis [18, 19]. MVTx or isolated small bowel transplantation is the treatment of choice for most of such patients.

The patients were transplanted because of different indications, but we divided the patients into three groups (Table 1): the indication for the first group who were transplanted for benign processes is internationally accepted. The second category included MVTx for surgical resection of slow-growing intra-abdominal tumors, is also accepted as the last choice for selected tumors otherwise unresectable in some centers. The last group is the most challenging indication. There is no general consensus among transplant community for the third group of our patients with locally advanced intra-abdominal malignancies and there is no consensus if visceral transplantation is an indication for this group of patients or not. 

Nephrotoxicity is one of the most serious complications observed after transplantation. Renal damage is the result of pre-existing renal dysfunction in some of these patients as well as a side effect of calcineurin inhibitors. Biological agents such as ATG and alemtuzumab (Campath 1H) not only decrease the chance of developing nephrotoxicity but also have a proven role in long-term survival of both graft and the patient [11, 20, 21]. We used this strategy and all the patients received a biological agent as induction immunosuppression before transplantation in our series. 

Post-transplant lymphoproliferative disorder (PTLD), which develops as a result of over-immunosuppression, is a common cause of late graft loss and mortality, especially after intestinal transplantation [22, 23]. Like PTLD, GVHD is also more common after intestinal transplantation than any other solid organ transplants, most likely due to the large number of donor cells in intestinal grafts. Unlike other series [10, 24], we observed only one case of GVHD and no one with PTLD and recurrence of disease, probably because of the small number of patients in our series and short-term follow-up. 

The overall survival in our series was 57% after a median follow-up of 8.7 months, which is not much different from other reports [10, 12]. All of the survivors have tolerated oral feeding and were not dependent on TPN therapy any more. 

Eight patients in our series died after transplantation. The cause of death in five of them was septicemia. The main sources of sepsis were intra-abdominal including collections, anastomotic leakage and jejunostomy tube complications. There were different risk factors that predisposed the patients to major infections. Prolonged operative time (needed for tumor resection, retroperitoneal lymph node dissection and the implantation phase), excessive blood loss and transfusions, prolonged ischemic time of the grafts, potent immunomodulators like alemtuzumab and ATG with resultant leukopenia and inevitable multiple intestinal anastomoses in a patient with edematous and inflamed small intestine, were among the main reasons for serious infections in our series. 

In conclusion, MVTx and small bowel transplant become acceptable treatment options for benign abdominal pathologies. However, we would not recommend it for intra-abdominal malignancies. Infection and septicemia are still major life-threatening complications in small bowel and MVTx. 
